# 

**Published:** 1868-05

**Authors:** 


### CONTENTS:

### ORIGINAL COMMUNICATIONS.

PAGE.

ARTICLE I.—Artificial Palates. By Dr. Wilhelm Suersen, Sen.......... 1

ARTICLE II.—On the Physiological Action of the Muscles which control and influence the Lower Jaw. By Thomas Brian Gunning, M.D.,D.D.S.......... 10

### CORRESPONDENCE.

Dental College Commencements.......... 22

Answers to Querists.—Treatment of Teeth rendered sensitive by Mechanical Abrasion.......... 23

Extracting Teeth for Pregnant Females.......... 24

### SELECTED ARTICLES.

ARTICLE III.—On the Surgery of the Hand. By Stephen Smith, M.D.......... 25

ARTICLE IV.—Examination of Blood-Stains.......... 29

ARTICLE V.—Staphylorrhapy in the case of Congenital Fissure of the Hard and Soft Palate. By R. P. Howard, M.D., L.R.C.S.E., etc.......... 32

### MONTHLY SUMMARY.

How to Extract Silver from Lead.......... 36

On the Characteristics of the Different Varieties of Creosote.......... 37

To Make Linen Fire-Proof.......... 38

Antidote for Strychnia.......... 38

Excision of Breast; Anæsthesia by Nitrous Oxide Gas.......... 38

Sulphuric Acid in Living Amimals.......... 39

Ozone.......... 40

Absinthism.......... 40

Causation of Phthisis.......... 40

Digitalis.......... 41

Tumors of the Tongue and Pharynx.......... 41

Peripheral Termination of the Motor Nerves.......... 42

Origin and Nature of Movement.......... 43

Influence exerted by Anæsthetics on the Brain and Nervous System.......... 44

Influence of Electricity on Muscular Fibre of Vegetable Life and on Nutrition.. 46

New Method of Local Anæsthesia, Applicable to the Extraction of teeth and other Operations in the Cavity of the Mouth.......... 47

### BIBLIOGRAPHICAL NOTICES.

Plastic Surgery.......... 48

### EDITORIAL DEPARTMENT.

The Association of the Colleges of Dentistry.......... 49

A Degree Easily Obtained.......... 49

Oxy-Chloride of Zinc over Exposed Nerves.......... 50

The Action of the Philadelphia Dental College.......... 51

Prof. Charles E. Dunn.......... 52

